# Chitosan Ameliorates Candida auris Virulence in a Galleria mellonella Infection Model

**DOI:** 10.1128/AAC.00476-20

**Published:** 2020-07-22

**Authors:** Laís Salomão Arias, Mark C. Butcher, Bryn Short, Emily McKloud, Chris Delaney, Ryan Kean, Douglas Roberto Monteiro, Craig Williams, Gordon Ramage, Jason L. Brown

**Affiliations:** a Oral Sciences Research Group, Glasgow Dental School, School of Medicine, College of Medical, Veterinary and Life Sciences, University of Glasgow, Glasgow, United Kingdom; b São Paulo State University (Unesp), School of Dentistry, Department of Preventive and Restorative Dentistry, São Paulo, Brazil; c Department of Biological and Biomedical Sciences, School of Health and Life Sciences, Glasgow Caledonian University, Glasgow, United Kingdom; d Glasgow Biofilm Research Network, Glasgow Dental School, Glasgow, United Kingdom; e Graduate Program in Dentistry, University of Western São Paulo (UNOESTE), Prudente/São Paulo, Brazil

**Keywords:** *Candida auris*, chitosan, therapeutics, aggregates, *Galleria mellonella*

## Abstract

Candida auris has emerged as a multidrug-resistant nosocomial pathogen over the last decade. Outbreaks of the organism in health care facilities have resulted in life-threatening invasive candidiasis in over 40 countries worldwide. Resistance by C. auris to conventional antifungal drugs such as fluconazole and amphotericin B means that alternative therapeutics must be explored. As such, this study served to investigate the efficacy of a naturally derived polysaccharide called chitosan against aggregative (Agg) and nonaggregative (non-Agg) isolates of C. auris *in vitro* and *in vivo. In vitro* results indicated that chitosan was effective against planktonic and sessile forms of Agg and non-Agg C. auris. In a Galleria mellonella model to assess C. auris virulence, chitosan treatment was shown to ameliorate killing effects of both C. auris phenotypes (NCPF 8973 and NCPF 8978, respectively) *in vivo*. Specifically, chitosan reduced the fungal load and increased survival rates of infected *Galleria*, while treatment alone was nontoxic to the larvae. Finally, chitosan treatment appeared to induce a stress-like gene expression response in NCPF 8973 in the larvae likely arising from a protective response by the organism to resist antifungal activity of the compound. Taken together, results from this study demonstrate that naturally derived compounds such as chitosan may be useful alternatives to conventional antifungals against C. auris.

## Abstract (precis)

Candida auris has emerged as a multidrug-resistant nosocomial pathogen over the last decade. Outbreaks of the organism in health care facilities have resulted in life-threatening invasive candidiasis in over 40 countries worldwide. Resistance by C. auris to conventional antifungal drugs such as fluconazole and amphotericin B means that alternative therapeutics must be explored. As such, this study served to investigate the efficacy of a naturally derived polysaccharide called chitosan against aggregative (Agg) and nonaggregative (non-Agg) isolates of C. auris *in vitro* and *in vivo. In vitro* results indicated that chitosan was effective against planktonic and sessile forms of Agg and non-Agg C. auris.

## INTRODUCTION

Fungal diseases are highly prevalent; nearly a billion people worldwide are estimated to have skin, nail, and hair fungal infections (1). Of these diseases, health care-associated fungal infections are commonplace. Recently, Candida auris has gained unprecedented attention due to its emergence as a prolific nosocomial pathogen. Since its first discovery in 2009 (2), the organism has reportedly been identified in over 40 countries on 6 different continents, with a crude mortality rate of 66% associated with C. auris candidemia (3). Coupled with the alarmingly high multidrug resistance profile in C. auris, this organism provides a substantial global risk in health care facilities and intensive care units (4–6). In addition, the organism has the ability to persist environmentally, with suggestions that its emergence has coincided with climate change based on its particular attributes (7). *In vitro* studies have shown that standard and high-level strategies of disinfection are incapable of completely eradicating C. auris off of nonporous surfaces (8, 9), while cellular aggregates of C. auris can survive for as long as 14 days even following treatment with clinically relevant concentrations of sodium hypochlorite (10). As such, identification of new antifungal therapies is of utmost importance.

Whole-genome sequencing of C. auris originally led to the identification of four geographically and phylogenetically distinct clades of the organism, each containing genetically identical strains with vast (>100,000) single nucleotide polymorphism (SNP) differences between clades (4). Recently, a fifth clade has been proposed, separated from other clades by >200,000 SNPs (11). Within these clades exist C. auris isolates that have two distinguishable phenotypes, aggregative (Agg) and nonaggregative (non-Agg) isolates (12). In the former, a characteristic accumulation of aggregates containing yeast cells attached to daughter cells after budding are visible *in vitro*, which cannot be physically disrupted. Furthermore, such aggregates have recently been isolated from harvested tissues of murine models infected with C. auris, suggestive that such a phenotype can be observed *in vivo* (13). *In vitro*, the non-Agg phenotype, which is characterized by sparse, individual cellular entities, forms biofilms with greater biomass than Agg counterparts (14). In a Galleria mellonella killing assay, non-Agg C. auris isolates were significantly more virulent than the aggregate-forming isolates, resulting in increased larvae death (12, 14). The identification of these unique C. auris isolates from various clades further complicates antifungal susceptibility testing, particularly given the differences in virulence traits between the two phenotypes.

Chitosan (poly-(β-1→4)-2-amino-2-deoxy-d-glucopyranose) is a naturally occurring, biodegradable, and nontoxic polysaccharide derived from deacetylated chitin (a constituent of fungal cell walls and crustacean exoskeletons), with wide-spectrum antimicrobial activity against different microorganisms (15). As such, the antimicrobial polymer compound provides an exciting alternative to conventional antibacterial and/or antifungal therapeutics. Indeed, several studies have investigated the antifungal efficacy of chitosan or derivatives against a range of *Candida* species, including Candida albicans and other clinically relevant fungal species (16–20). At this juncture, the aim of this study was to test the ability of chitosan against non-Agg and Agg isolates of C. auris *in vitro*. Furthermore, chitosan efficacy was then tested against two candidate C. auris isolates *in vivo* (one non-Agg isolate, NCPF 8973, and one Agg isolate, NCPF 8978) in a *Galleria* infection model.

## RESULTS

Given the well-established drug resistance profile of C. auris (4), we sought to assess the antimicrobial potential of chitosan, a polymer purported to have broad-spectrum activity. We assessed the activity of chitosan against a selection of C. auris isolates with different phenotypes, both *in vitro* and *in vivo*. Firstly, the MICs for planktonic (PMIC) and sessile (SMIC) cells were determined for a total of eight different isolates of C. auris (four non-Agg and four Agg isolates, as shown in Table 1). All isolates used possessed similar sensitivity profiles to conventional antifungals as based on sensitivity profiles reported previously (Table 1) (12, 14). PMIC values varied between 5 and 20 mg/liter chitosan for all isolates, with the highest PMIC (20 mg/liter) observed for two Agg isolates (NCPF 8977 and NCPF 8978). It is noteworthy that planktonic forms of these two isolates have previously been shown to be highly resistant to caspofungin (14). The SMIC_50_ and SMIC_80_ values also varied between non-Agg and Agg phenotypes of C. auris. For SMIC_50_, these values ranged between 10 and 80 mg/liter, and for SMIC_80_, they ranged from 40 to 160 mg/liter. The highest SMIC_80_ was detected in the same two Agg isolates as above (160 mg/liter for NCPF 8977 and NCPF 8978). Interestingly, there was a certain level of heterogeneity observed in the non-Agg and Agg isolates response to chitosan treatment irrespective of aggregative phenotype.

**TABLE 1:** Planktonic and sessile MICs of chitosan against eight isolates of Candida auris^*a*^

Phenotype	Strain (characteristics)^*b*^	Clade	PMIC (mg/liter)	SMIC_50_ (mg/liter)	SMIC_80_ (mg/liter)
Non-Agg	C. auris 8973^*c*^ (FluR, AmpI, EchS)	Southern Asian/Indian	5	40	40
Non-Agg	C. auris 8989 (FluR, AmpI, EchS)	Southern Asian/Indian	10	40	80
Non-Agg	C. auris 8971 (FluR, AmpI, EchI)	Southern Asian/Indian	10	40	80
Non-Agg	C. auris 199 (FluR, AmpI, EchS)	South African	10	10	80
Agg	C. auris 8977 (FluR, AmpI, EchS)	South African	20	40	160
Agg	C. auris 8978^*c*^ (FluR, AmpI, EchS)	South African	20	80	160
Agg	C. auris 8983 (FluR, AmpI, EchS)	Southern Asian/Indian	5	40	40
Agg	C. auris 8986 (FluR, AmpI, EchS)	Southern Asian/Indian	10	20	40

a Planktonic (PMIC) and sessile MICs (SMIC) for chitosan against four nonaggregative (non-Agg) and four aggregative (Agg) isolates of C. auris. For PMICs, the broth microdilution method was employed. For SMICs, the XTT metabolic reduction assay was used, and the SMIC_50_ and SMIC_80_ corresponds to the concentration that resulted in 50% and 80% reduction of XTT readings when compared to those of the untreated positive control. All MIC tests were performed on 2 independent occasions, showing identical results each time. For comparative purposes, conventional antifungal susceptibility profiles are shown for all eight isolates as assessed by broth microdilution method and as previously described (12, 14).

b FluR, fluconazole resistant; AmpI, amphotericin B intermediate; EchI or EchS, echinocandins intermediate or susceptible (51).

c Two isolates selected for microscopic analyses and Galleria mellonella infection model.

**FIG 1:**
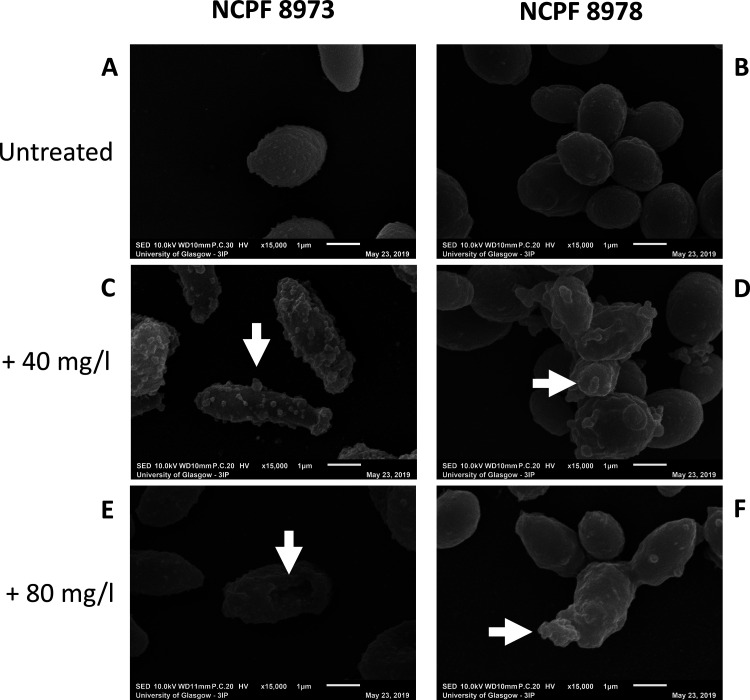
Scanning electron microscopic images of chitosan-treated Candida auris. Chitosan-treated 24-h biofilms of nonaggregative (non-Agg) NCPF 8973 and aggregative (Agg) NCPF 8978 C. auris were visualized using scanning electron microscopy (SEM). (A and B) Untreated non-Agg and Agg biofilms were used as controls and treated in the same way minus chitosan. Non-Agg and Agg biofilms of C. auris were treated with 40 mg/liter (C and D) or 80 mg/liter (E and F) for 24 h prior to imaging at ×15,000 magnification. White arrows highlight the encapsulation of C. auris cells by chitosan particles and deflation in cell morphology of the non-Agg NCPF 8973 isolate (E).

Next, 24-h chitosan-treated C. auris biofilms were visualized using scanning and transmission electron microscopy (SEM and TEM) to assess the ultrastructure and morphology of the cells after treatment. For these and subsequent studies, one non-Agg (NCPF 8973) isolate and one Agg (NCPF 8978) isolate were selected for analysis. In SEM, at ×15,000 magnification, morphological differences in the sessile C. auris cells were observed after exposure to chitosan. Untreated non-Agg C. auris biofilms displayed singular oval-shaped yeast cells (Fig. 1A), while the Agg phenotype of C. auris resulted in clusters or “aggregates” of oval-shaped yeast cells (Fig. 1B). At both concentrations of chitosan (40 and 80 mg/liter), the drug can be seen coating and encapsulating C. auris cells in the biofilms (Fig. 1C to F). Intriguingly, the higher concentration of chitosan appeared to adsorb to the cell surface and puncture the non-Agg C. auris cell, resulting in a deflated appearance likely resulting from cell death (Fig. 1E, white arrow). At the same concentration, chitosan can be seen coating the Agg C. auris cell, however, with no obvious change in morphology (Fig. 1F). Such a discrepancy in ultrastructure between the two phenotypes may correlate with the differences in MICs as shown in Table 1, whereby 80 mg/liter was two times the SMIC_80_ for the non-Agg C. auris NCPF 8973.

**FIG 2:**
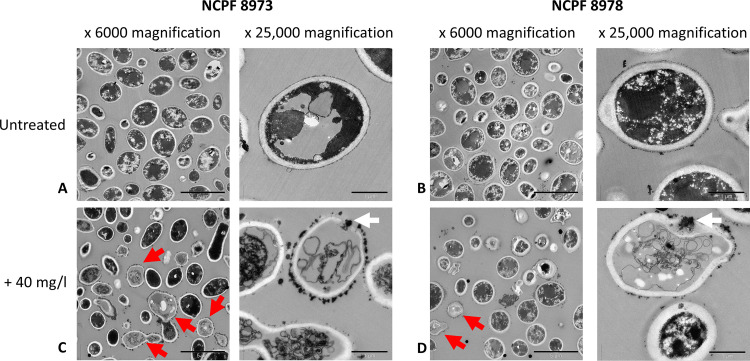
Transmission electron microscopic images of chitosan-treated Candida auris. Planktonic cells of nonaggregative (non-Agg) NCPF 8973 and aggregative (Agg) NCPF 8978 C. auris at 1 × 10^8^ cells/ml were treated with chitosan prior to imaging using transmission electron microscopy (TEM). (A and B) Untreated non-Agg and Agg controls were used as comparison minus chitosan treatment. (C and D) Non-Agg and Agg C. auris were treated with 40 mg/liter of chitosan for 24 h prior to TEM imaging at ×6,000 and ×25,000 magnification. Red arrows in lower magnification panels identify the coating of C. auris cell walls with chitosan particles. White arrows in higher magnification panels highlight penetration of C. auris cell wall/membranes by chitosan.

Further analyses into the interactions between chitosan and C. auris were achieved using TEM. TEM images showed that chitosan particles coated the cells of both C. auris isolates (Fig. 2). Untreated cells for non-Agg and Agg C. auris appear darkened, with dense intracellular material and a thick, uniform cell wall (Fig. 2A and B). Following treatment with 40 mg/liter of chitosan, particles of the compound are seen coating the cell walls/membranes of both isolates (denoted by red arrows in Fig. 2C and D). At higher magnification, accumulation of chitosan is evident in the periphery of the cell, leading to penetration of the cell wall and membrane (white arrows in Fig. 2C and D, right panels). Interestingly, chitosan-coated cells for both isolates appear transparent with evidence of a loss of cell morphology and/or cell components, potentially resulting from an efflux of intracellular material following penetration of the cell by the compound.

**FIG 3:**
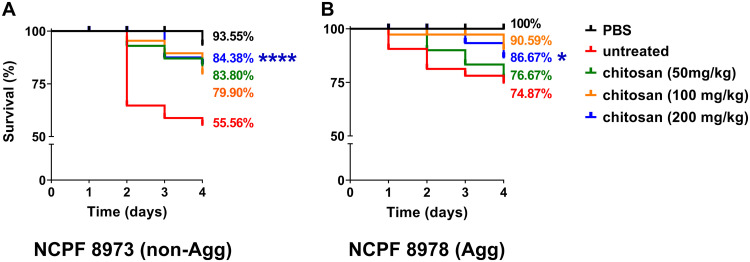
Survival curves of Galleria mellonella following infection with Candida auris. G. mellonella larvae were infected with 2.5 × 10^5^ cells/larvae of C. auris ± chitosan treatment, and survival rates were monitored every 24 h for 4 days. A total of 3 chitosan treatments were used (50 mg/kg, green line; 100 mg/kg, yellow line; and 200 mg/kg, blue line). Control groups received PBS only (black line) or were infected with C. auris minus chitosan treatment (red line). The highest concentration of chitosan had no effect on the survival of the larvae minus C. auris infection (data not shown). Data representative of results from three independent experiments with 10 larvae per group are shown in a Kaplan-Meier plot, and statistical differences are calculated between treatment groups by the log rank (Mantel-Cox) test. *, Significant differences between highest concentration of chitosan (200 mg/kg) and infected controls minus chitosan treatment (*, *P* < 0.05; ****, *P* < 0.0001).

Given the differences in Agg and non-Agg C. auris responses to chitosan *in vitro*, the efficacy of chitosan was next tested *in vivo* in a G. mellonella infection model. Firstly, the virulence of non-Agg and Agg C. auris was assessed in the model using a Kaplan-Meier plot to monitor the survival of infected G. mellonella larvae over 4 days postinfection. Similar to previous studies, the non-Agg NCPF 8973 isolate was significantly more virulent than the Agg NCPF 8978 isolate. Infection with NCPF 8973 resulted in killing of ∼45% of the larvae 4 days postinfection, while following infection with NCPF 8978, almost 80% of the larvae remained alive (Fig. 3). Following treatment with chitosan, the compound ameliorated the killing effects of both C. auris isolates in the infection model. After treatment, at the highest concentration, 200 mg of chitosan/kg of body weight significantly increased the survival rates of the larvae infected with NCPF 8973 from ~55% to ~84% (Fig. 3A; ****, *P* < 0.0001 according to log rank test). For the Agg phenotype, in comparison to those of the the untreated group, survival rates of Agg C. auris-infected larvae were significantly increased to ∼87% from ∼75% when treated with 200 mg/kg chitosan (Fig. 3B; *, *P* < 0.05).

**FIG 4:**
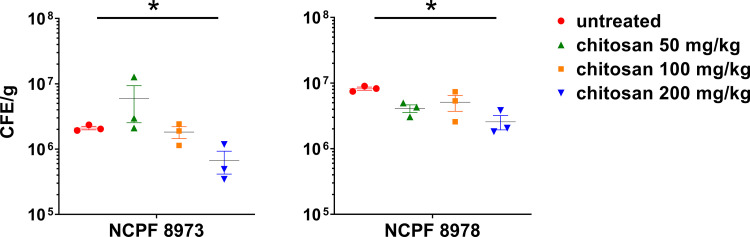
Fungal load from Candida auris-infected Galleria mellonella. G. mellonella larvae were infected with 2.5 × 10^5^ cells/larvae of C. auris ± chitosan treatment. After 24 h, larvae were harvested and weighed prior to DNA extraction. The abundance of C. auris (presented as colony forming equivalents per gram) in the larvae was calculated by quantitative PCR using a standard curve methodology of fungal CFU ranging from 1 × 10^3^ to 1 × 10^8^ CFU/ml. Significant differences were calculated using a one-way analysis of variance (ANOVA) with Tukey’s posttest. Significant differences denoted by an asterisk (*, *P* < 0.05). Data representative of results from three independent experiments.

**FIG 5:**
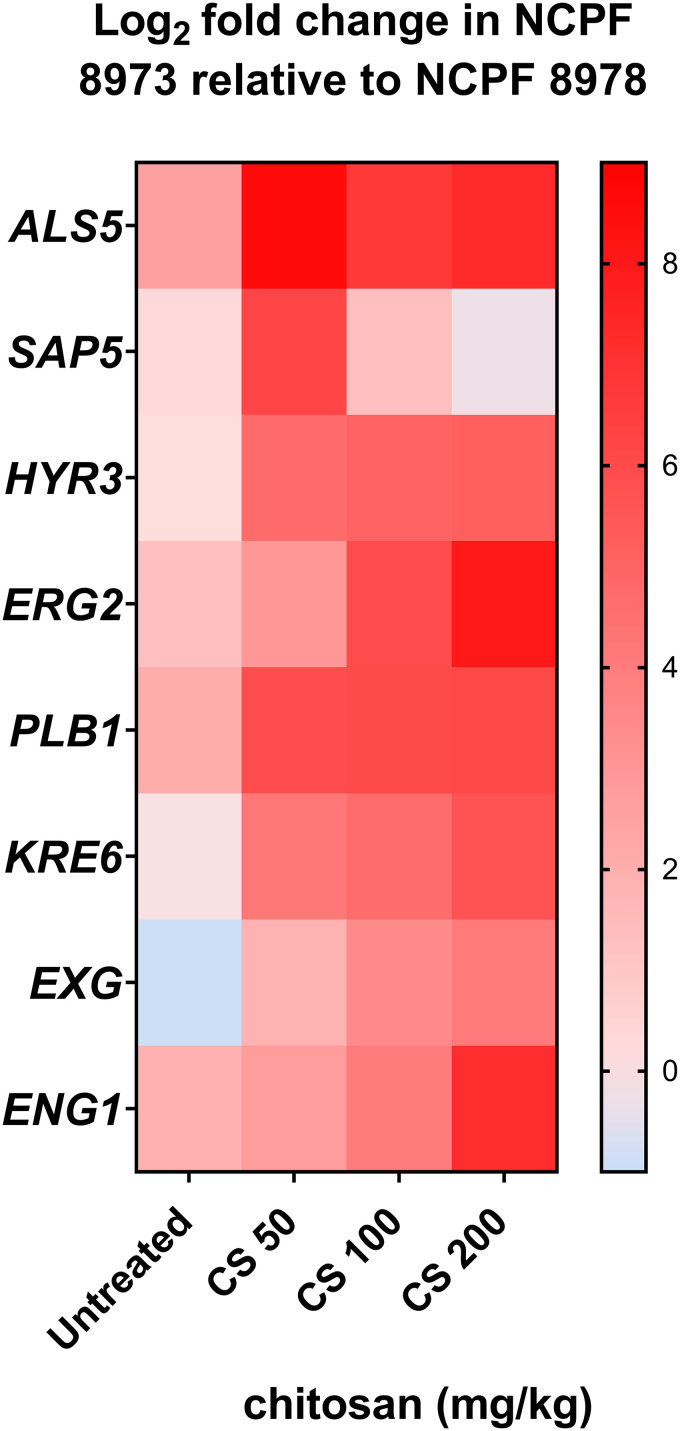
Gene expression profile of Candida auris in the Galleria mellonella infection model. G. mellonella larvae were infected with 2.5 × 10^5^ cells/larvae of C. auris ± three concentrations of chitosan treatment (50 mg/kg, 100 mg/kg, and 200 mg/kg). After 24 h, larvae were harvested for RNA extraction. Gene expression was measured by quantitative PCR, and expression of all genes of interest was calculated relative to a housekeeping gene (β-*actin*). Data were presented as mean values from three independent experiments in a heatmap, calculated as log_2_ fold change of expression in C. auris NCPF 8973 relative to C. auris NCPF 8978 ± chitosan treatment.

In order to provide mechanistic insights behind the observed protective effect of chitosan *in vivo*, fungal load and C. auris gene expression was determined in the infected larvae ± chitosan treatment. Firstly, fungal load in larvae was significantly reduced following treatment with chitosan (200 mg/kg) for both non-Agg and Agg infection models (Fig. 4; *, *P* < 0.05). For gene expression analyses in the fungi, expression of genes related to adhesion (*ALS5*, *HYR3*), hydrolytic enzymes (*SAP5*, *PLB1*), cell wall, cell membrane, and extracellular matrix (*ERG2*, *KRE6*, *EXG*, *ENG1*) were investigated in relation to the housekeeping gene *β-actin*. These candidate genes were selected for analyses as previously described; these and similar genes are differentially regulated in early and mature biofilms of C. auris (21). Expression of all genes arrayed (with the exception of *SAP5*) were upregulated in the non-Agg C. auris isolate when presented as log_2_ fold change relative to the Agg isolate (Fig. 5). All genes were upregulated in the non-Agg isolate following treatment with the highest concentration of chitosan compared to those of the untreated controls (see Fig. S1 in the supplemental material, white bars). The greatest change in expression was seen for *ALS5*, changing from ∼0.49% average expression in untreated controls to ∼13.02% average expression following treatment with 200 mg/kg chitosan (see Fig. S1B). For the Agg isolate, *SAP5* was the only gene upregulated following treatment with the highest chitosan concentration (Fig. S1C) (∼1.18% in the untreated controls compared to ∼21.88% for 200 mg/kg). *EXG* and *ENG1* were the most downregulated genes; expression changed from 8.92% and 6.41% in controls to 0.98% and 0.27% for 200 mg/kg in chitosan-treated isolates. Taken together, following treatment, chitosan appeared to induce a stress-like response in the non-Agg isolate compared to that in the Agg isolate (Fig. 5).

## DISCUSSION

Given the propensity for C. auris to be resistant to a wide range of azoles, polyenes, and echinocandins (4, 22), alternative treatment methods need to be explored. Here, a naturally derived compound called chitosan was shown effective against Agg and non-Agg isolates of C. auris both *in vitro* and *in vivo*. Recently, the compound was shown to be effective against C. albicans and other *Candida* species (16–19); the proposed mechanism of action is that positively charged chitosan molecules interact with negatively charged cell membranes leading to release of proteinaceous and intracellular constituents, causing cell death (15, 23, 24). Here, we were able to show that Agg and non-Agg C. auris planktonic and sessile cells were susceptible to chitosan treatment *in vitro*. Using microscopic analyses, the chitosan can be visualized coating the cell surface of the C. auris, resulting in an altered morphological phenotype likely arising from cell death. In addition, C. auris fungal load was reduced and its virulence ameliorated *in vivo* in a *Galleria* infection model following treatment with the compound. Interestingly, chitosan treatment induced a stress-like gene response in the more susceptible non-Agg isolate infected in the larvae.

C. auris isolates possess unique strain-specific variability in biofilm formation and virulence (10, 12, 14). Two types of C. auris isolates exist, one that forms an aggregative phenotype *in vitro* and the second that forms single-cell biofilms (12, 14). Therefore, studies must account for these differences in aggregative phenotype when studying the effects of potential therapeutics against C. auris. As such, this study initially tested the susceptibility of four Agg and non-Agg isolates of C. auris to chitosan. Interestingly, C. auris response to chitosan exhibited a level of heterogeneity in the Agg and non-Agg isolates. These observations are in line with a previous study showing variation in planktonic and sessile MICs for a number of C. auris isolates taken from intensive care unit or candidemic patients against a wide range of conventional antifungal therapies, such as amphotericin B, fluconazole, and caspofungin (25). Given the high level of heterogeneity among isolates to different antifungals, future work merits consideration for direct comparative studies testing novel therapeutics and conventional antifungals against isolates with different phenotypes from various clades.

MIC testing results indicated that two isolates, NCPF 8973 and NCPF 8978, had the lowest and highest PMIC and SMIC_80_, of the initial pool, respectively. Therefore, we hypothesized that these two isolates would provide accurate representations for the effects of chitosan against the Agg and non-Agg phenotypes. For these two isolates, the PMIC and SMIC values were higher for the Agg phenotype. This finding may be explained by the Agg phenotype of C. auris providing a protective barrier to therapeutics. Indeed, we have recently shown that Agg C. auris NCPF 8978 possesses the ability to survive and persist on surfaces in response to sodium hypochlorite treatment, even after 14 days posttreatment. Conversely, the non-Agg isolate, C. auris NCPF 8973, was susceptible to such treatment (10). Here, it was evident from SEM imaging that the non-Agg C. auris is visibly encapsulated by the chitosan compound leading to an altered morphology at the higher concentration of drug, while in the aggregative phenotype, there appears to be no change in cellular ultrastructure, suggestive of a protective phenotype when cells are present in aggregates. The altered morphology in the non-Agg isolate SEM images likely arose from chitosan-mediated cell death. In agreement, a publication by Ganan et al. recently used confocal microscopic imagery to show that an oligosaccharide of chitosan (chitooligosaccharide) generated from chemical or enzymatic digestion of chitosan was capable of adsorbing to yeast cells of C. albicans, subsequently disrupting cellular structure and accumulating in the cytoplasm (17). Indeed, TEM images confirmed that chitosan, albeit at a concentration above the PMIC for both isolates (e.g., 40 mg/liter), was visualized coating the organism and penetrating the cell walls, resulting in a loss of cellular morphology and efflux of intracellular material. Moving forward, it would be of interest to quantify the level of adsorption and cell wall/membrane damage in the C. auris isolates following treatment with the antifungal. However, such techniques as SEM and TEM are qualitative in nature, with no accurate way of quantifying the level of cellular damage.

In the *Galleria* model to assess C. auris virulence, the non-Agg C. auris NCPF 8973 induced significantly greater killing of the larvae than the Agg isolate, NCPF 8978. This is in agreement with previous studies from our group and others (12, 14), although why such phenomena arise is unknown. It could be postulated that single cellular forms of C. auris can disseminate more rapidly *in vivo* than Agg isolates, leading to increased killing rates. Others have shown that the phenotypic form of C. auris can switch from yeast to filamentous morphology following “passage” through a mammalian body, suggestive that phenotypic state is inducible under certain conditions (26). It would be of interest to assess whether such phenomena occur in G. mellonella larvae infected with non-Agg C. auris, which could explain its enhanced virulence traits in this model. Nevertheless, we were able to show that chitosan treatment ameliorated the C. auris virulence in this infection model, likely arising from reduced fungal load in the larvae. In a similar manner to the results presented here, several research groups have recently reported the use of novel antifungal compounds against C. auris *in vitro* and *in vivo* (27–29). However, such *in vivo* studies are generally limited to studying individual isolates or isolates with similar Agg or non-Agg phenotypes. Such Agg or non-Agg characteristics need to be considered, particularly given that similar murine infection models have shown that C. auris aggregates can accumulate in tissues of infected animals (13). Therefore, we deemed it pertinent to study the effects of possible antifungal therapeutics against different C. auris aggregates *in vivo*. However, it must be stated that the *in vivo* observations described here are limited to one non-Agg isolate and one Agg isolate. Therefore, assumptions about the effects of the chitosan on other isolates in similar model systems cannot be made without further studies, particularly given the high level of heterogeneity among isolates to the antifungal.

Finally, differential gene expression of the Agg and non-Agg C. auris was observed in the *Galleria* model with chitosan treatment. Several candidate genes involved in important virulence pathways associated with biofilm formation and resistance were selected for comparative expression analyses in the isolates. Although such analyses were limited to one isolate for each phenotype, results were indicative of a unique stress-like response in the non-Agg phenotype following treatment with chitosan *in vivo*, evident by an upregulation in expression of most of the genes arrayed. In a similar manner, others have identified that chitosan can interfere in gene expression in other *Candida* species. In addition to chitosan penetrating the cell wall and membrane of the cell leading to cell death (15, 23, 24), it is postulated that the compound is able to breach the nucleus of the fungal cell interfering with the synthesis of mRNA and translation of proteins (30). An *in vitro* study recently showed that chitosan represses the function of the SAGA (Spt-Ada-Gcn5 acetyltransferase) complex in C. albicans by downregulating *ADA2* and associated genes, which are involved in encoding for proteins involved in maintaining cell wall and membrane integrity (16). Conversely, it could be postulated that the stress-like response of NCPF 8973 to chitosan may simply have resulted from an increased susceptibility to the compound (as shown by the *in vitro* MIC tests). Conclusive elucidation of the mechanistic response seen in C. auris to chitosan is currently unknown and requires further investigation.

Direct physical interactions between chitosan and the cell wall may provide an alternative mechanism by which the drug affects gene expression in C. auris. It is not uncommon for antifungal therapies to induce such stress-like responses in *Candida* species, particularly *in vitro* (31, 32). For example, echinocandins, such as caspofungin, which target β-glucan synthesis pathways can exert stress upon the cell wall leading to attenuated efficacy against C. albicans at high concentrations (33). Similar drug resistance profiles have recently been described for C. auris to echinocandins, resulting from a mutation *FKS1* gene, which encodes for a 1-3-β-glucan synthase enzyme (34). Interestingly, in our analyses, we found an upregulation in genes associated with cell wall component assembly/disassembly and cell separation pathways in the non-Agg C. auris in increasing concentrations of chitosan. In particular, the genes *KRE6* (involved in β-1,6-glucan synthesis), *EXG*, and *ENG1* (exo‐β‐1,3‐glucanase and endo-β-1,3-glucanase, involved in cell separation) were upregulated in the non-Agg phenotype following treatment of the *Galleria* larvae with the drug. Such genes have been shown to be important in virulence of other fungal species. For example, disrupting the β-1,6-glucan synthesis pathway by targeting *KRE6* and a related gene *SKN1* reduced growth and biofilm-forming rates of C. albicans, interfered with cell separation and cell wall formation, and attenuated its virulence in a murine model (35). Furthermore, mutation of *ENG1* impaired virulence of *Histoplasma* yeasts *in vivo* (36). Therefore, it could be postulated that the gene expression profiles observed in this study may be indicative of a response by the organism to upregulate cell wall β-glucan synthesis and cell separation in an attempt to promote survival and circumvent the antifungal effect of chitosan.

Similar gene expression responses were not seen in the Agg isolate of C. auris following treatment with chitosan. Such a result is difficult to interpret without further studies on this and other Agg isolates. However, *in vitro* observations from this study showed that the Agg isolate was more resistant to chitosan treatment than the non-Agg counterpart, which could explain the observed gene expression profile *in vivo*. As discussed above, the aggregative phenotype may simply provide a physical barrier for chitosan delivery to the cell. Indeed, this aggregative phenomenon may exist both *in vitro* and *in vivo*. Ben-Ami and colleagues recently recovered large aggregates of C. auris cells from the harvested tissue of a murine model following infection, which could be a strategy used to evade the host response (13). Nevertheless, the clinical implications of aggregation in C. auris remain limited, although such phenomena have been considered for other microorganisms. The formation of aggregates in bacteria such as Pseudomonas aeruginosa enhances tolerance traits such as antibiotic resistance and/or evasion of the host response (37, 38). It would be of great interest to assess whether such C. auris isolates can form aggregates in *Galleria* tissues and whether non-Agg isolates persist as single cells *in vivo*. If achievable, this could begin to elucidate the resistance mechanisms utilized by C. auris against antifungals.

In conclusion, this is the first study to show that the naturally derived molecule chitosan may be effective against the putative opportunistic environmental yeast, C. auris. We and others have shown here and in previous studies that the aggregative phenotypes of different C. auris isolates dictate the response of the organism to antifungals (8, 9, 12, 14). As such, future studies must continue to investigate these unique aggregative phenotypic traits of C. auris isolates from different clades to fully comprehend the response of such isolates to conventional and novel therapeutics.

## MATERIALS AND METHODS

### Microbial growth.

The four Agg and four non-Agg isolates of C. auris that were used in this study were kindly gifted by Andy Borman (Public Health England). The eight isolates and their clades are shown in Table 1. These isolates were taken from various clinical sites (Public Health England National Collection of Pathogenic Fungi [NCPF]) as previously described (12, 39). The isolates were deemed aggregative if they could not be physically disrupted by vigorous vortex mixing or by detergent treatments as previously described (12).

All isolates were stored in Microbank beads (Pro-Lab Diagnostics, UK) and then grown at 30°C for 24 to 48 h. All isolates were maintained on Sabouraud (SAB) dextrose agar (Oxoid, Hampshire, UK) at 4°C prior to propagation in yeast-peptone-dextrose (YPD; Sigma-Aldrich, Dorset, UK) medium overnight (16 h) at 30°C, gently shaking at 200 rpm. Cells were pelleted by centrifugation (3,000 × *g*) and then washed two times in phosphate-buffered saline (PBS). Fungal cells were then standardized to desired concentration after counting using a hemocytometer in appropriate media as described below.

### Planktonic and sessile susceptibility testing with chitosan.

Chitosan used throughout this study was purchased from Sigma-Aldrich (medium molecular weight, 75 to 85% deacetylated; catalog number 448877). Chitosan stocks of 1.4 g/liter were freshly prepared in 2% acetic acid, constantly stirring (200 rpm) for 24 h at room temperature until complete solubilization as previously described (40, 41). All subsequent studies were then completed in accordance with the minimum information guidelines specified for planktonic and/or biofilm testing in microplates (42). Where appropriate, Clinical and Laboratory Standards Institute (CLSI) guidelines were followed for all planktonic susceptibility tests (43).

First, the broth microdilution method (43) was used to determine the MIC of chitosan on planktonic C. auris cells (PMICs). In short, yeast cells were standardized to 1 × 10^4^ CFU/ml in RPMI 1640 medium. These were then inoculated in 96-well round-bottom plates (Corning, Flintshire, UK) containing serial double dilutions of chitosan (ranging from 0.68 to 350 mg/liter), and PMICs were visually determined after 24 to 48 h. For comparative purposes, conventional antifungal (fluconazole, amphotericin B, and echinocandins [micafungin and caspofungin]) PMIC testing was also done for all eight isolates as assessed by broth microdilution method in a similar manner as described above.

For biofilm (sessile) MICs (SMICs), the XTT (2,3-bis-(2-methoxy-4-nitro-5-sulfophenyl)-2H-tetrazolium-5-carboxanilide salt [Sigma-Aldrich, Dorset, UK]) metabolic reduction assay was used as described elsewhere (44). In brief, biofilms were formed by culturing yeast cells at 1 × 10^6^ CFU/ml in RPMI medium in flat-bottom wells of 96-well plates (Corning, UK) for 24 h at 37°C prior to treatment for an additional 24 h. The SMIC_50_ and SMIC_80_ corresponds to the concentration that resulted in 50% and 80% reduction of XTT readings when compared to the untreated positive control. All MIC tests were carried out on two separate occasions in quadruplicate wells of a 96-well plate. For all experiments, appropriate negative controls minus inoculum were included on each plate in quadruplicate.

### Galleria mellonella infection model.

Two isolates of C. auris (one non-Agg isolate, NCPF 8973, and one Agg isolate, NCPF 8978) were selected for the Galleria mellonella killing assay as previously described (14). G. mellonella larvae were infected with these two isolates in the presence and absence of chitosan in a similar manner to that described elsewhere (45–47). In short, 10 sixth instar G. mellonella larvae (Livefoods Direct Ltd, Sheffield, UK) weighing between 200 and 300 mg were selected for each test group. For infection, a 50-μl Hamilton syringe equipped with a 26-gauge needle was used to inject C. auris into the *Galleria* larvae; 10 μl of C. auris (2.5 × 10^5^ cells/larva) were inoculated through the hindmost right proleg of each larva. The infected larvae were placed in sterile petri dishes and incubated at 37°C for 2 h. After this period, larvae were injected in the last left proleg with chitosan at different concentrations (50, 100, and 200 mg/kg). Larvae inoculated with PBS and the highest dose of chitosan alone (e.g., 200 mg/kg) were also included as controls. The percentage survival of the larvae was monitored every 24 h over 4 days. A larva was considered dead when it displayed no movement in response to touch together with a dark discoloration of the cuticle. The experiment was repeated on three separate occasions with 10 larvae per group.

### RNA extraction and differential gene expression analysis in C. auris.

RNA was extracted from infected and uninfected larvae as follows. Three larvae from each experimental group were snap frozen in liquid nitrogen and ground to a fine powder by mortar and pestle in TRIzol prior to bead beating with 0.5-mm glass beads using a BeadBug microtube homogenizer for a total of 90 s (Benchmark Scientific, NJ, USA). RNA was then extracted using the RNeasy minikit according to the manufacturers’ instructions (Qiagen Ltd, Crawley, UK) and quantified using a NanoDrop 1000 spectrophotometer (Thermo Scientific, UK). RNA was converted to cDNA using the High-Capacity RNA-to-cDNA kit (Life Technologies, Paisley, UK) as per the manufacturer’s instructions. Gene expression of C. auris infected in the larvae was determined using quantitative PCR (qPCR) from a total of three individual larvae, each taken from three independent experiments. For qPCR analyses, the StepOnePlus PCR machine was used with the following PCR thermal profiles: holding stage at 50°C for 2 min, followed by denaturation stage at 95°C for 10 min and then 40 cycles of 95°C for 3 s and 60°C for 15 s. Expression levels of each gene of interest were calculated using the ΔΔ threshold cycle (ΔΔCT) method (48), with expression normalized to housekeeping gene β-*actin*. qPCR gene expression data were presented as percent expression relative to housekeeping gene or log_2_ fold change in untreated and treated NCPF 8973 relative to NCPF 8978. All primer sequences used for qPCR are shown in Table 2.

**TABLE 2:** List of primer sequences used in this study^*a*^

Study	Gene	Direction	Sequence (5′–3′)
Fungal load quantification	*ITS*	Forward	TCGCATCGATGAAGAACGCAGC
		Reverse	TCTTTTCCTCCGCTTATTGATATGC
Gene expression studies	β-*actin*	Forward	GGCTCATCTTGGCTTCCTT
		Reverse	GGACCGGACTCGTCGTATTC
	*SAP5*	Forward	GGATGCAGCTCTTCCTGGTT
		Reverse	CTTCCAGTTTGCGGTTGTGG
	*PLB1*	Forward	TGCCATCTACAACCCGAACC
		Reverse	TCAACGACGACAAGGGAAGG
	*ENG1*	Forward	TGTGAAGGATGAGGCTGCTG
		Reverse	GTGCTAGTCACACCACCGAA
	*ERG2*	Forward	ACACAAAGCCGAATGGCAAC
		Reverse	GAGAGGCCAAGTGAAGCAGT
	*ALS5*	Forward	ATACCAGGGTCGGTAGCAGT
		Reverse	CTATCTTCGCCGCTTGGGAT
	*HYR3*	Forward	TTCGACTTCCCTGAGCCAAC
		Reverse	AGCTCGAAACAGCAGACGAA
	*KRE6*	Forward	ATCACGATCGACATGGGCTC
		Reverse	TCAACGACAACGAAAACGGC
	*EXG*	Forward	CAACAAAGGCGTCAACTGGG
		Reverse	TTCATCCACAGGGACAGTGC

a Forward and reverse primer sequences for C. auris fungal load quantification and gene expression analyses in this study.

### DNA extraction for calculating fungal burden in *Galleria* model.

Uninfected and infected *Galleria* larvae were processed as above for RNA extraction before a back-extraction buffer (50 mM sodium citrate, 4 M guanidine thiocyanate, and 1M Tris [pH 8.0]) was used to extract DNA from samples as previously described (45). Colony-forming equivalents (CFE) of C. auris were then determined using *ITS* gene primers (sequences shown in Table 2) by qPCR and CFE per milliliter quantified using a standard curve methodology of fungal CFU. Briefly, CFU of C. auris NCPF 8973 and NCPF 8978 that equate to 1 × 10^3^ to 1 × 10^8^ cells/ml were determined using a hemocytometer prior to DNA extraction. DNA extracted from 1 × 10^3^ to 1 × 10^8^ cells/ml of C. auris was used for quantitative analyses using qPCR to generate a standard curve. All samples, including standards were run in duplicate for qPCR analyses. Fungal load was calculated from a total of three individual infected larvae each taken from three independent experiments.

### Scanning and transmission electron microscopy.

For scanning electron microscopy (SEM), non-Agg C. auris (NCPF 8973) and Agg C. auris (NCPF 8978) were grown as described above. Biofilms were then prepared as described above by growth in RPMI 1640 medium on 13-mm Thermanox coverslips (Fisher Scientific, Loughborough, UK) placed in the bottom of 24-well microtiter plates (Corning, UK) for 24 h at 37°C. After incubation, biofilms were treated with chitosan at 40 or 80 mg/liter (diluted in RPMI 1640 medium) for an additional 24 h and then prepared for scanning electron microscopy (SEM) as previously described (49). In brief, following incubation, chitosan-treated and untreated biofilms were washed three times with PBS prior to fixation in a solution containing 2% glutaraldehyde, 2% paraformaldehyde, 0.15% alcian blue, and 0.15 M sodium cacodylate (pH 7.4). Biofilms were then sputter coated with gold and viewed under a JEOL JSM-6400 scanning electron microscope (JEOL Ltd, Hertfordshire, UK). All SEM images included in this study were captured at ×15,000 magnification.

For transmission electron microscopy (TEM), samples were prepared as follows. Non-agg C. auris (NCPF 8973) and Agg C. auris (NCPF 8978) were grown in YPD as described above and then standardized to 1 × 10^8^ cells/ml in PBS. One-milliliter aliquots of each isolate were transferred to Eppendorf tubes and treated for an additional 24 h, planktonically, with chitosan at 40 mg/liter diluted in RPMI 1640 medium. Posttreatment, the samples were centrifuged at 13,000 × *g* for 5 min, supernatants discarded, and pellet retained for TEM and prepared for imaging as previously described (50). Following preparation, samples were embedded in araldite/Epon 812 resin and sectioned using a Leica ultracut UCT and Diatome diamond knife. Samples were imaged on a JEOL 1200 EX TEM (JEOL Ltd, Hertfordshire, UK) running at 80 kV. All TEM images included in this study were captured at ×6,000 or ×25,000 magnification.

### Statistical analysis.

Statistical analyses were performed using GraphPad Prism (version 8; GraphPad Software Inc., La Jolla, CA). Two-tailed Student’s *t* tests were used to compare the means of two samples or one-way analysis of variance (ANOVA) to compare the means of more than two samples. Tukey’s posttest was applied to the *P* value to account for multiple comparisons of the data. Where appropriate, statistical tests on qPCR gene expression data were completed on ΔCT values. Pooled data from three independent experiments of G. mellonella larvae killing assay were assessed using the Kaplan-Meier method, and treatment groups were compared by the log rank (Mantel-Cox) test. *P* values of <0.05 were considered statistically significant for all tests.

## Supplementary Material

Supplemental file 1
