# Supplementary material for: Chitosan Ameliorates Candida auris Virulence in a Galleria mellonella Infection Model
Source: Antimicrob Agents Chemother. 2020 Jul 22;64(8):e00476-20. doi: 10.1128/AAC.00476-20 (PMC7526850; doi:10.1128/AAC.00476-20)
Supplement: Supplemental file 1 [file AAC.00476-20-s0001.pdf]

# Chitosan ameliorates *Candida auris* virulence in a *Galleria mellonella* infection model

**Laís Salomão Arias[1,2], Mark C Butcher[1,4], Bryn Short[1,4], Emily McKloud[1,4], Chris Delaney[1,4], Ryan Kean[3,4], Douglas Roberto Monteiro[2], Craig Williams[1,4], Gordon Ramage[1,4*], Jason L Brown[1,4*]**

## Supplementary Figure legends

**Figure 1 – Percentage gene expression of Agg and non-Agg *Candida auris* in the *Galleria mellonella* infection model.** *G. mellonella* larvae were infected with 2.5 x $10^5$ cells/larvae of Agg and non-Agg *C. auris* +/- three concentrations of chitosan treatment (50 mg/kg, 100 mg/kg and 200 mg/kg). After 24 h, larvae were harvested for RNA extraction and gene expression was measured by quantitative PCR, and % expression of all genes of interest presented relative to a house-keeping gene (*β-actin*) (*C. auris* NCPF 8973; white bars, C. auris NCPF 8978; grey bars).

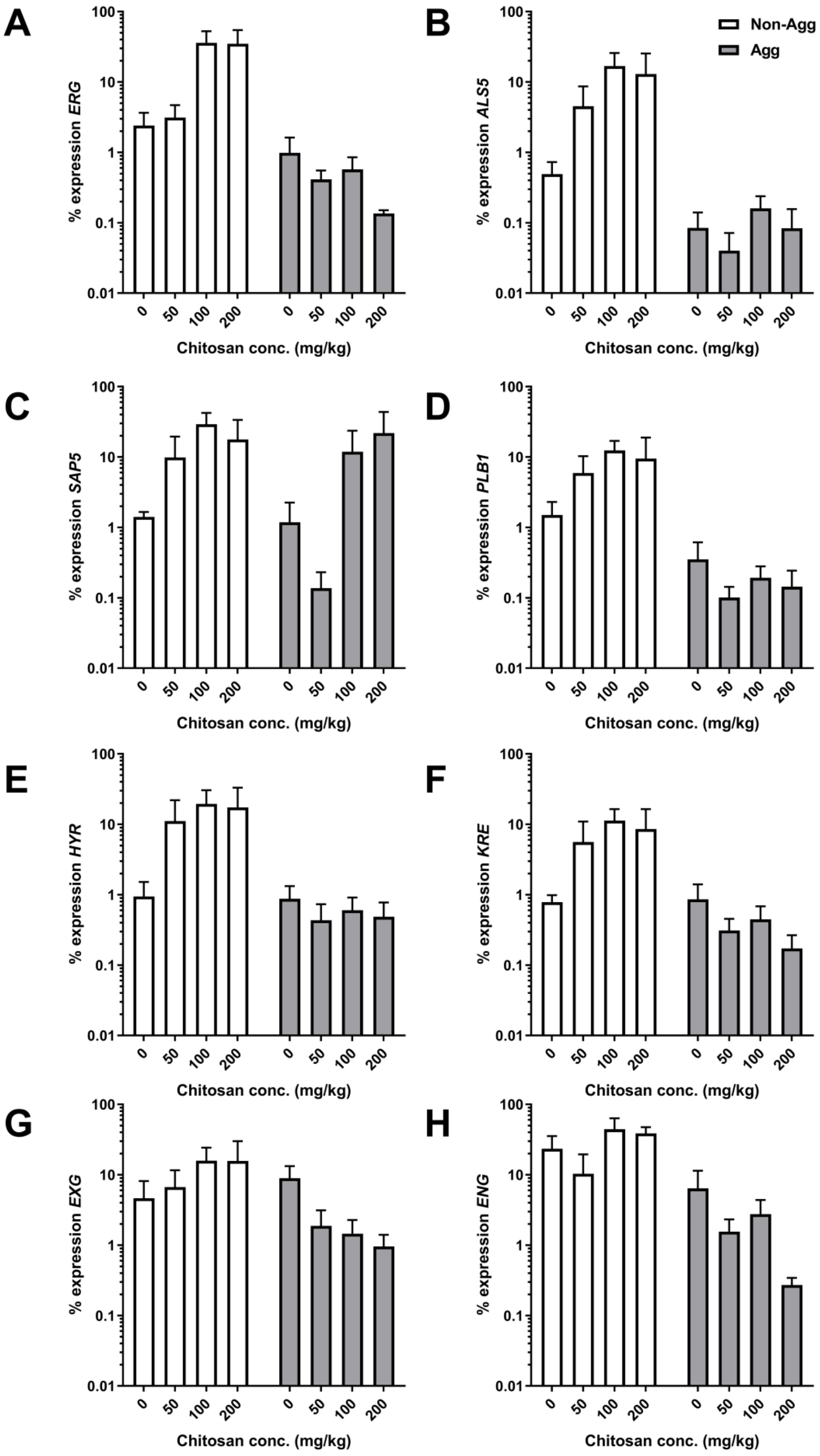
A
B
C
D
E
F
G
H
Non-Agg
Agg
% expression ERG
% expression ALS5
% expression SAP5
% expression PLB1
% expression HYR
% expression KRE
% expression EXG
% expression ENG
100
10
1
0.1
0.01
0
50
100
200
Chitosan conc. (mg/kg)
